# Mortality of New York in 1848

**Published:** 1849-05

**Authors:** 


### MORTALITY OF NEW YORK IN 1848.

The city inspector of New York has made his annual report of deaths in that city, from which it appears that the total number for 1848 was 15,919. This number includes 1,161 prematurely born, and 205 brought to the city for interment, which deducted leaves the mortality 14,553. Of this number 327 died of casualties, suicide, and murder.

Of the deaths reported there were 7,020 adults, and 8,899 children; of whom 8,343 were males, and 7,576 females; 11,302 were natives of the United States, 3,949 of Ireland, 694 of Germany, 454 of England, 141 of Scotland, and 68 of France. In 1847 the mortality was 14,441, showing that the increase of population has not been attended with a corresponding increase of mortality, Excluding casualties, the excess of deaths in 1848 over the previous year was only 27. The greatest mortality in 1847 was from typhus fever, the number of deaths being 1,396. In 1848 the mortality from the same cause was but 943. The number of deaths from diseases of the bowels was 2,227; from small pox 444, an increase of 301 over the preceding year, which the inspector attributes to the neglect of those precautions which have been urged upon the citizens by the proper authorities. Among the causes of death may be noticed 12 cases of poisoning, 7 from hydrophobia, 20 tetanus, and 34 suicides.

